# Deciphering the microbiome–metabolome landscape of an inflammatory bowel disease inception cohort

**DOI:** 10.1080/19490976.2025.2527863

**Published:** 2025-07-18

**Authors:** Shiva T. Radhakrishnan, Benjamin H. Mullish, Marton L. Olbei, Nathan P. Danckert, Maria A. Valdivia-Garcia, Jose I. Serrano-Contreras, Despoina Chrysostomou, Sharmili Balarajah, Robert W. Perry, John P. Thomas, Lejla Potari-Gul, Dezso Modos, Lucy C. Hicks, Nick Powell, Timothy R. Orchard, Jia V. Li, Julian R. Marchesi, Tamas Korcsmaros, James L. Alexander, Horace R. T. Williams

**Affiliations:** a Division of Digestive Diseases, Department of Metabolism, Digestion and Reproduction, Faculty of Medicine, Imperial College London, London, UK; b Departments of Gastroenterology and Hepatology, St Mary’s Hospital, Imperial College Healthcare NHS Trust, London, UK; c UKRI MRC Laboratory of Medical Sciences, Hammersmith Hospital Campus, London, UK; d Quadram Institute Bioscience, Norwich Research Park, Norwich, UK; e Division of Systems Medicine, Department of Metabolism, Digestion and Reproduction, Faculty of Medicine, Imperial College London, London, UK; f NIHR Imperial BRC Organoid Facility, Imperial College London, London, UK

**Keywords:** Microbiome, Metabolome, IBD, Network analyses, Inception cohort, Crohn‘s Disease, Ulcerative Colitis

## Abstract

The gut microbiota contribute to the etiopathogenesis of inflammatory bowel disease (IBD), but limitations of prior studies include the use of sequencing alone (restricting exploration of the contribution of microbiota functionality) and the recruitment of patients with well-established disease (introducing potential confounders, such as immunomodulatory medication). Here, we analyze a true IBD inception cohort and healthy controls (HCs) via stool 16S rRNA gene sequencing and multi-system metabolomic phenotyping (using nuclear magnetic spectroscopy and mass spectroscopy), with subsequent integrative network analysis employed to delineate novel microbiota–metabolome interactions in IBD. Marked differences in β diversity and taxonomic profiles were observed both between IBD and HCs, as well as between Crohn’s disease (CD) and ulcerative colitis (UC) patients. Multiple between-group metabolomic differences were also observed, particularly with regard to tryptophan-/indole-related metabolites; for example, UC patients had higher levels of serum metabolites including xanthurenic acid (*q* = 0.0092) and picolinic acid (*q* = 0.018). Network analysis demonstrated multiple unique interactions in CD compared to HCs with minimal overlap, indicating a loss of ‘health-associated’ interactions in CD. Compared to HCs, UC patients demonstrated increased pathway activity related to nitrogen and butanoate metabolism, whilst CD patients displayed increased leucine and valine synthesis. Networks from IBD patients overall showed negative correlation with health-specific associations, including an increase in taurine metabolism. Collectively, this work characterizes multiple novel perturbed microbiota–metabolome interactions that are present even at the diagnosis of IBD, which may inform potential future targets to aid diagnosis and direct therapeutic options.

## Introduction

Inflammatory bowel diseases (IBD) are chronic, relapsing-remitting conditions primarily affecting the gastrointestinal (GI) tract. The two main subtypes of IBD are Crohn’s disease (CD) and ulcerative colitis (UC). Although the etiopathogenesis of IBD development is yet to be fully understood, a combination of environmental, genetic, and immunological factors appears to play important roles.^1^ The gut microbiota play a crucial role in the etiopathogenesis and clinical trajectory of IBD, with IBD patients exhibiting an over-representation of traditionally pro-inflammatory microbes in addition to an overall reduction in microbial diversity.^2^ Pro-inflammatory bacteria are thought to adversely impact upon the mucus barrier layer of the bowel, a proposed mechanism of intestinal inflammation.^3,4^ Prior data document a reduction in α diversity within the gut microbiome of patients suffering from terminal ileal Crohn’s disease (CD) when compared to healthy controls (HCs).^5^ However, when comparing patients with ulcerative colitis (UC) to those with CD, such significant differences in α diversity have not been observed.^5^

Previous studies have characterized the gut microbiota in the context of IBD; however, most of these studies have focused on patients who have been previously diagnosed with IBD and have been exposed to numerous previous treatments.^5,6^ It is therefore difficult to ascertain if the “microbiota fingerprint” associated with clinical IBD phenotypes is a cause, a consequence (e.g., related to medication use), or an epiphenomenon.

Studies leveraging multi-omics approaches, notably metabolomics, have revealed that changes in microbial composition are closely linked with shifts in the production of microbial metabolites, many of which have immunomodulatory properties.^7,8^ Furthermore, recent data have shown that a multi-omics approach outperforms single-omic analyses in predicting outcomes in IBD using machine learning.^9^

Analyses of ‘newly diagnosed’ and untreated IBD cohorts are limited, but those that do exist have yielded interesting results. Multi-omics approaches have revealed that patients with CD characterized by “dysbiosis” exhibit a marked reduction in butyrate and secondary bile acids (BAs) such as lithocholate and deoxycholate compared to those with milder inflammation.^8^ However, this study primarily involved pediatric patients, and there were instances where some CD patients did not contribute baseline samples. Reduced concentrations of SCFAs have been shown in UC patients^10^; an increased urinary concentration of xanthurenic acid has also been associated with severe UC.^11^ In another study, 31 microbial species and 13 metabolites were found to effectively distinguish IBD from HCs.^12^ These studies have also highlighted the difficulty of obtaining unadulterated samples from a treatment-naïve population. One study included patients with previous surgical resection for IBD; therefore, this was not a truly treatment-naïve cohort^10^; another included sample collection after initial colonoscopy,^12^ which may have affected results, as bowel purgatives may impact the gut microbiota and metabolite composition.^13,14^

Tryptophan (TRP) metabolites have generated great interest in IBD: the gut microbiota are known to be important in the metabolism pathways of TRP.^15^ Previous data have shown an increased serum concentration of quinolinic acid in IBD patients compared to healthy controls,^16^ with a recent publication showing mucosal inflammation was associated with reduced concentrations of xanthurenic and kynurenic acid.^17^

Network biology analyses have previously been used by large clinical cohorts to integrate microbiome data with other data modalities – including metabolomics, metagenomics, transcriptomics, and clinical data – to help build hypotheses about disease mechanisms.^8^ Theoretically, interrogating personalized networks in individual patients could reveal novel biomarkers of interest in addition to therapeutic targets that would be individualized to each patient.^18^

There have been very few studies documenting microbial composition and functionality in newly diagnosed IBD patients, where samples were obtained prior to any endoscopic intervention, formal diagnosis, or (most importantly) treatment. Here, we present the gut microbial composition and functionality of an inception cohort of IBD prior to formal diagnosis. We find distinctive microbiome and metabolomic signatures in a true inception IBD cohort that are different from established IBD cohorts and integrate these data in network biology analyses.

The aims of this study were to generate novel potential insights into IBD pathogenesis by:

1. Documenting differences in the gut microbiome and metabolome between treatment-naïve newly diagnosed CD, UC patients, and healthy controls, using samples obtained before diagnosis.
2. Undertaking integrated microbiome-metabolome network-level analyses by focusing on those interactions that were lost or gained when comparing CD, UC, and healthy controls.

## Materials and methods

### Study participants and sample collection

Patients with symptoms suggestive of IBD, but no formal diagnosis, were consecutively recruited in a single Gastroenterology unit in London, United Kingdom. Patients were excluded if they had a prior diagnosis of IBD. Healthy controls (HCs) were also consecutively recruited independently – these were healthy individuals with no medical conditions and on no regular medication.

Research ethics approval was obtained (REC reference 18/LO/1207, protocol number 18SM4548, IRAS ID 243,310). Both HCs and patients were excluded if antibiotics were administered within the previous three months.

Demographic and clinical data and biofluid samples were collected prospectively prior to any treatment, bowel preparation, endoscopy, or formal diagnosis. Whole fecal samples were collected with a FECOTAINER^Ⓡ^ fecal collection device.^19^ Serum samples were obtained by venesection with subsequent centrifugation and were stored in microcentrifuge tubes. Midstream urine samples were collected in universal containers; urinary sediment was separated using centrifugation and urine was stored in microcentrifuge tubes. Samples were also collected from HCs. All samples collected from patients and HCs were immediately stored at 4°C, for a maximum of 30 minutes, prior to processing and storage at −80°C. Participant phenotypic data were collected including age, weight, height, ethnicity, sex, and dietary information. To document IBD disease severity, the Harvey-Bradshaw index (HBI) (for CD) and simple clinical colitis activity index (SCCAI) (for UC) were recorded.^20–22^ Fecal samples were processed for fecal calprotectin (FC) values in recruited patients, prior to endoscopic evaluation as part of routine clinical care, with the range of quantification 20–20000 μg/g. FC was measured using an Abbott Architect c800 analyzer, with reagent, calibration and control used as BUHLMANN FCAL turbo.

### DNA extraction, metataxonomic sequencing, and bacterial biomass quantification

DNA was extracted from crude fecal samples using the DNeasy PowerLyser PowerSoil Kit (Qiagen, Hilden, Germany) using the manufacturer’s established protocols. An additional step was undertaken to homogenize samples using Bullet Blender Storm bead beater (Chembio, St Alban’s, UK). The V1-V2 hypervariable regions of the 16S rRNA gene were amplified for microbiota taxonomic identification (see Appendix 1a within the Supplementary for primer sequences). Total microbial biomass within each sample was calculated using quantitative real-time polymerase chain reaction (qPCR) analyses. BactQuant assay was chosen given the comprehensive coverage of actual bacterial DNA, although not necessarily live microbes.^23^ qPCR analyses were undertaken to enable transformation of compositional metataxonomic data into ecosystem abundance,^24^ removing the need for rarefaction.^25^ Sequence libraries were prepared using 2x300bp Illumina MiSeq chemistry following the Illumina’s 16S Metagenomic Sequencing Library Preparation Protocol.^26^

### Nuclear magnetic resonance spectroscopy (^1^H NMR) methods

To analyze fecal and urine samples, buffer (created using previously detailed protocols^27^) and samples were combined in a 1:9 ratio (buffer:sample) for final analyses. Serum analyses were undertaken using previously described protocols.^27^ All biofluid samples were prepared for analyses as per previous protocols.^28^ Sample extracts were analyzed with a Bruker 600 MHz AVANCE II ^1^H-NMR spectrometer at 300 K. 1D ^1^H NMR spectra were acquired using a standard one-dimensional pulse sequence, with saturation of the water resonance (noesygppr1d pulse program) during both the relaxation delay (RD = 4 s) and mixing time (tm = 10 ms). In total, 4 dummy scans, 128 scans, and 64 K data points were collected; 2D spectra were also obtained to increase confidence in metabolite identification. Further information regarding the acquisition of 2D spectra and other aspects concerning ^1^H NMR parameters is given in Appendix 2 within the Supplementary Material.

### Liquid chromatography mass spectroscopy (LC-MS) methods

Fecal water was prepared from lyophilized stool as per previously published protocols.^29^ Urine and serum LC-MS experiments were undertaken at the National Phenome Centre (NPC) and followed previously described protocols^29,30^ to obtain targeted and global profiles for the serum, urine, and fecal samples. Targeted assays included tryptophan (TRP) metabolites and bile acids (BA). To identify and quantify fecal and serum short chain carboxylic acids (SCCAs), including short chain fatty acids (SCFAs), previously published protocols were followed.^31^ Global analyses were completed with fecal reverse phase (RP) negative and positive phases in addition to urinary RP negative phase.

### Data processing

16S rRNA gene amplicon sequencing data processing was carried out using the DADA2 pipeline (v.1.18)^32^ with Silva taxonomic database (v138.1).^33^ The mean sequencing depth per sample was 36,325, after DADA2 quality processing; additional details regarding sequencing depth are given in Appendix 1b within the Supplementary Material. Data were analyzed using R studio environment to construct the phylogenetic tree using the Phangorn package with default settings.^34^ A suite of R packages were used to analyze the microbiome sequencing data including Phyloseq,^35^ Vegan,^36^ and ggplot2.^37^

^1^H-NMR data were analyzed in SIMCA® (version 17, Sartorius, Sweden) and MATLAB (2014a, MathWorks) to visualize the data. The individual spectra of these metabolites were identified, as previously described, after cross-referencing internal and external databases, including the Human Metabolome Database (HMDB) and the biological Magnetic Resonance Data Bank.^38–40^

Untargeted LC-MS data were processed using “peakPantheR”, an established R package to identify metabolites as previously described,^41^ these identified metabolites, including respective PubCHEM IDs, are detailed in Appendix 6 within the Supplementary Material. LC-MS data were processed using the online platform MetaboAnalyst (version 5.0).^42^

### Statistical and network analyses

Kruskal–Wallis test was used to study the significance of differences in α diversity, taxonomic abundance, and metabolite concentration between cohorts. Between-group alpha diversity comparison was also performed using linear mixed effects models with disease type (i.e., UC, CD), age, sex, BMI, ethnicity, diet, and smoking status as covariates; no random effect was included. Regarding β diversity, sequencing data were first transformed using a centered log-ratio (CLR) and absolute abundance was used with qPCR data as microbiome datasets are compositions (as previously described).^24^ Subsequently, spatial organization in study groups was analyzed in the CLR transformed data with a non-metric multidimensional scaling (NMDS) plot based on Aitchson’s distance to account for the data’s compositional nature. Using a permutational multivariate analysis of variance (PERMANOVA; at 999 permutations), with the adonis2 package in R,^36^ we compared microbial β diversity (as before, adjusting for disease type (i.e., UC, CD), age, sex, BMI, ethnicity, and smoking status). Statistically significant results are reported on PCA plots (i.e., adjusted p-value). Homogeneity of dispersion was analyzed using betadisper and permutest functions in the R vegan package.^36^ Benjamini–Hochberg false discovery rate (FDR) adjustment was used for *p* value correction where multiple comparisons were performed.^43^

To ascertain which metabolites were responsible for the differences observed, loading plots for each orthogonal projection to latent structures’ discriminant analysis (OPLS-DA) were created in MATLAB^Ⓡ^ which enabled identification of the metabolites at extreme ends of the loading plot, driving the differences noted. Semi-targeted ^1^H-NMR metabolite differences were compared across cohorts using column analyses after log-transformation. Kruskal–Wallis tests were used to compare the mean rank of each cohort with FDR *p* value correction.

The processed fecal metabolite and bacterial abundance data were read into R (version 4.4.1). The individual ASVs were aggregated to genus level. Genera not present in at least 20% of all patients were removed to ensure that any rare taxa or sequencing artifacts were removed from the analysis.

Microbial network analyses were formulated by calculating the Spearman correlation coefficient between the measured fecal metabolites and the bacterial amplicon sequencing variants (ASVs) for each disease state (UC, CD, Healthy), using the rcorr function from the Hmisc package (version 5.1) in R (version 4.4.1). Results were filtered for significance (FDR *p* < = 0.05) and magnitude (*r* > = |0.5|). Visualization was carried out with the ggplot,^37^ ggraph^44^ and tidygraph^45^ packages.

The corresponding bacterial ASVs were collapsed into their respective taxonomic genera to allow assessment of bacterial genus – metabolite associations. Functional enrichment analysis of the strongly correlating microbiota associated metabolites was carried out using the MetaboAnalyst web server.^46^ The enrichment ratio was computed by dividing the number of expected hits with the observed hits, as returned by MetaboAnalyst. For the analysis of matching associations with opposite correlations across states, the median of correlation values was calculated for each genus – metabolite pair (to capture the behavior of genera with multiple associating strains), and the results were visualized on a scatterplot (Supplementary Figure S1–3). KEGG pathway^47^ enrichment analysis of the appropriate anticorrelation clusters found on the scatterplot (i.e. Healthy ≥ 0.4 and CD ≤ −0.4) was carried out using the MetaboAnalyst web server (Supplementary Figure S4).

Corrected *p* values (*q* values) of <0.1 were deemed to be significant given the exploratory nature of these analyses, with previous comparable microbiota datasets using a *q* value of <0.1 as significant.^48^

Node rewiring capturing bacteria with the most variable interaction profiles was calculated using the DyNet app^49^ (version: 1.0) in Cytoscape^50^ (version: 3.10.) with default settings (undirected networks).

## Results

### Patient phenotypes

In total 80 participants were recruited, comprising 60 IBD (23 CD and 37 UC) patients, newly diagnosed with IBD using established biochemical, endoscopic, histological, and radiological criteria,^51^ and 20 HCs. Between HCs and patients with IBD, there were no significant differences in key demographic variables such as age, BMI, diet, ethnicity, sex, or smoking status (*p* > 0.05 in all cases). There was no significant difference in the median fecal calprotectin (FC) value between CD and UC patients. Additionally, the median disease activity index (DAI) score did not significantly differ between UC and CD patients – as measured by SCCAI score and HBI scores, respectively. These data are displayed in Table 1.

**Table 1.** Phenotypic characteristics of the inception cohort at baseline.

General Characteristics	CD	UC	HCs	*p* value
Number of participants	23	37	20	
Age range, (median)	18–66, (35)	18–75, (32)	26–54, (34)	0.713*
Sex (M/F)	(*M* = 13/F = 10)	(*M* = 16/F = 21)	(*M* = 12/F = 8)	0.403 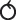
BMI range group (<18.5/18.6–24.9/>25.0) (kg/m^2^)	(5/13/5)	(6/22/9)	(1/18/1)	0.131 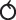
Diet (Meat eater/pescetarian/vegetarian)	(17/3/3)	(27/6/4)	(14/5/1)	0.790 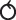
Ethnicity (Caucasian/non-Caucasian)	(12/11)	(26/11)	(12/8)	0.358 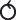
Smoking status (current/non-smoker)	(1/22)	(2/35)	(1/19)	0.983 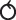
Fecal Calprotectin median, (range) (µg/g)	796, (259–2255)	1269, (258–7900)	N/A	0.146^∑^
DAI median, (range)	8, (4–14)	7, (4–15)	N/A	0.713^∑^

*KEY for statistical analyses used*: *=One way ANOVA; 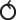
=Chi-square test; ∑ = Mann–Whitney U.

IBD patients were classified as per the Montreal classification.^52^ These data are displayed in Tables 2A and 2B.

**Table 2A.** UC cohort characteristics by Montreal classification.^52^

Extent of UC	E1	E2	E3
Number of participants*	7	16	14

*KEY*: *=One way ANOVA: FC value between phenotypes was not statistically significant (*p*=0.65).

**Table 2B.** CD cohort characteristics by Montreal classification.^52^

Crohn’s cohort demographics			
Location of CD*	L1= 10	L2= 4	L3= 9
Behavior of CD	B1= 16	B2= 3	B3= 4
Evidence of perianal disease	NO= 19	YES= 4	

*KEY*: *=One way ANOVA: FC value between phenotypes was not statistically significant (*p* = 0.40).

A total of 238 samples were analyzed: 78 fecal samples, 80 serum samples, and 80 urine samples.

### Gut microbiome diversity metrics and taxonomic features have a defined profile in an inception IBD cohort

**Figure 1.**
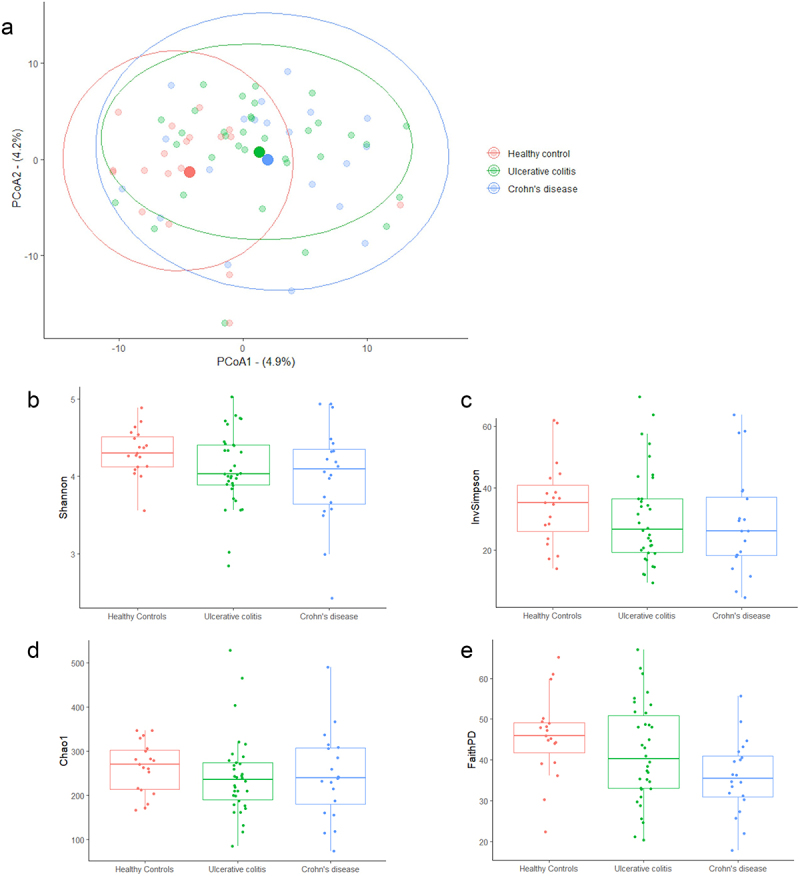
(a) PCoA of β diversity measurements HC vs UC vs CD (Ellipses representing 95% CI) *p*=0.018. Alpha diversity measurements HCs vs UC vs CD utilizing. (b) Shannon Index. (c) Inverse Simpson Index. (d) Chao 1 Index, E. Faith’s PD.(Median signified by middle horizontal line, 25% and 75% confidence intervals (CI) by box ends).

**Figure 2.**
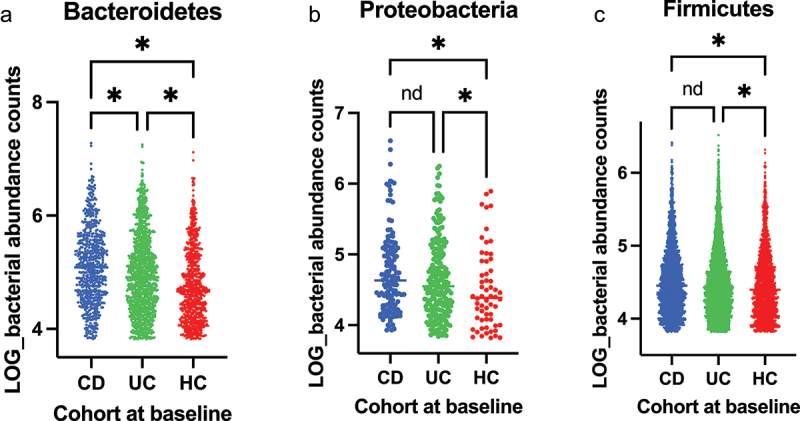
Median bacterial phyla count (qPCR data), HCs vs CD vs UC for (a) Bacteroidetes (ANOVA *p* < 0.001, CD vs HCs *q* < 0.001, UC vs HCs *q* < 0.001, CD vs UC *q* < 0.001). (b) Proteobacteria (ANOVA *p* = 0.01, CD vs HCs *q* = 0.0075, UC vs HCs *q* = 0.043, CD vs UC *q* = 0.16). (c) Firmicutes (ANOVA *p* = 0.001, CD vs HCs *q* < 0.001, UC vs HCs *q* = 0.011, CD vs UC *q* = 0.17). The level of significance is represented by: *<0.05, *nd*=no difference.

β diversity between the cohorts, as plotted by PCoA, illustrated that HCs were significantly different to both CD and UC populations (*p* = 0.0018) (Figure 1(a)). No significant difference in α diversity was demonstrated between CD and UC. Although the α diversity for HCs showed a trend of being increased compared to both UC and CD patients across multiple indices (Shannon, Inverse Simpson, Chao1, and Faith PD; Figure 1(b-d)), no between-group significance was found in a linear mixed-effects model that accounted for key demographic variables; specific α diversity statistical comparisons are detailed in Appendix 3 within the Supplementary Material. At the phylum level, the median abundance of Bacteroidetes (also known as Bacteroidota) was significantly different, with an overall ANOVA of *p* < 0.0001. Across inter-cohort comparisons, Bacteroidetes abundance was significantly different, with enrichment noted in CD compared to both HCs (*q* < 0.0001) and to UC (*q* < 0.0001). UC patients also demonstrated an increased median abundance of Bacteroidetes compared to HCs (*q* < 0.0001). The overall ANOVA for Firmicutes (also known as Bacillota) median abundance also reached significance with a *p* = 0.0011. Firmicutes were increased in both IBD cohorts compared to HCs, with CD > HCs (*q* = 0.0008) and UC > HCs (*q* = 0.011). Although the median abundance of Firmicutes was higher in CD compared to UC, this observation did not reach statistical significance (*q* = 0.17). Similar trends were observed in Proteobacteria (also known as Pseudomonadota) abundance with a significant overall ANOVA *p* = 0.0104. Proteobacteria median abundance was significantly higher in CD compared to HCs (*q* = 0.0075). This was also seen in the UC population who were enriched with Proteobacteria compared to HCs (*q* = 0.043). Key phyla differences are presented in Figure 2. Deeper analyses revealed that a particular Clostridia ASV from the Firmicutes phylum, identified as ASV455 by the Silvia database (part of the Family XIII AD3011 genus group), was enriched in HCs compared to both CD and UC patients (*q* < 0.001).

### Metabolomic profiles demonstrate distinct differences between controls and IBD in addition to between CD and UC

Principal component analyses (PCAs) on ^1^H NMR data demonstrated no outlying samples in fecal, serum, and urine analyses. OPLS-DA analyses comparing CD, UC, and HCs cohorts to each other revealed differences in metabolomic profiles between HCs and each IBD cohort, with crossvalidation analysis of variance (CV-ANOVA) confirming the significance of key models. Specifically, OPLS-DA analyses between serum samples of CD and UC demonstrated significant differences between the disease types (with a CV-ANOVA *p* = 0.028), but similar significant differences between CD and UC were not demonstrated in fecal and urine samples. These data are listed in Appendix 4 within the Supplementary Material.

**Figure 3.**
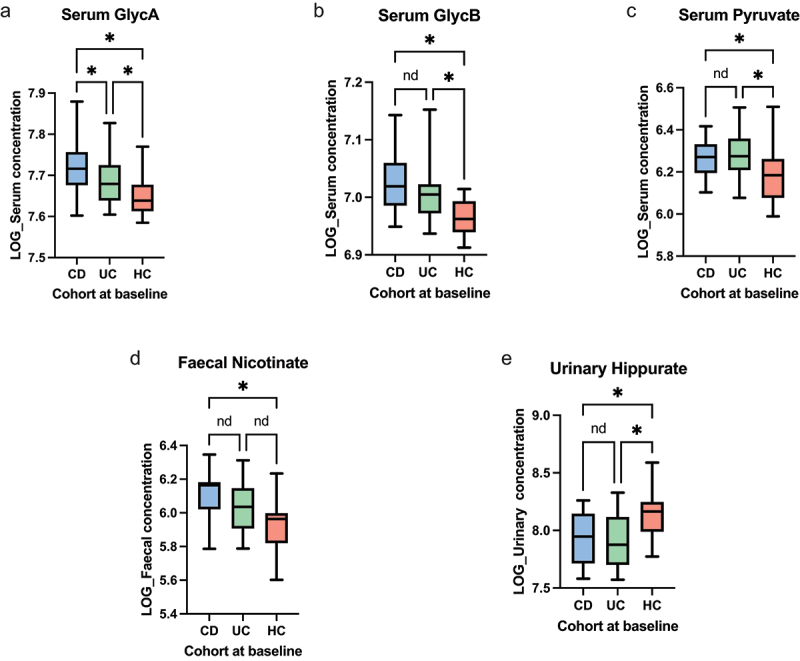
Univariate analyses of ^1^H-NMR data from inception cohort – (a) Serum GlycA (ANOVA *p* < 0.001, CD vs HCs *q* < 0.001, UC vs HCs *q* = 0.019, CD vs UC *q* = 0.048). (b) Serum GlycB (ANOVA *p* < 0.001, CD vs HCs *q* < 0.001, UC vs HCs *q* = 0.0017, CD vs UC *q* = 0.10). (c) Serum pyruvate (ANOVA *p* = 0.0079, CD vs HCs *q* = 0.028, UC vs HCs q = 0.0072, CD vs UC *q* = 0.64). (d) Fecal nicotinate (ANOVA *p* = 0.0047, CD vs HCs *q* = 0.0032, UC vs HCs *q* = 0.071, CD vs UC *q* = 0.083). (e) Urinary hippurate (ANOVA p < 0.001, CD vs HCs *q* = 0.0027, UC vs HCs *q* < 0.001, CD vs UC *q* = 0.74). The level of significance is represented by: *q<0.05, *nd*=no difference.

Semi-targeted ^1^H NMR analyses, with individual metabolite spectra identified using previously described databases,^38–40^ demonstrated that serum N-acetylglucosamine/galactosamine (GlycA) and sialic acid (GlycB) were higher in both CD and UC compared to HCs. Serum GlycA was also at a higher concentration in CD patients compared to UC patients. A similar trend was observed with serum pyruvate at a higher concentration in both IBD cohorts compared to HCs (Figure 3(a-c)). Fecal nicotinate was shown to be higher in CD patients (Figure 3(d)). Urinary hippurate was noted to be higher in HCs compared to both CD and UC (Figure 3(e)).

**Figure 4.**
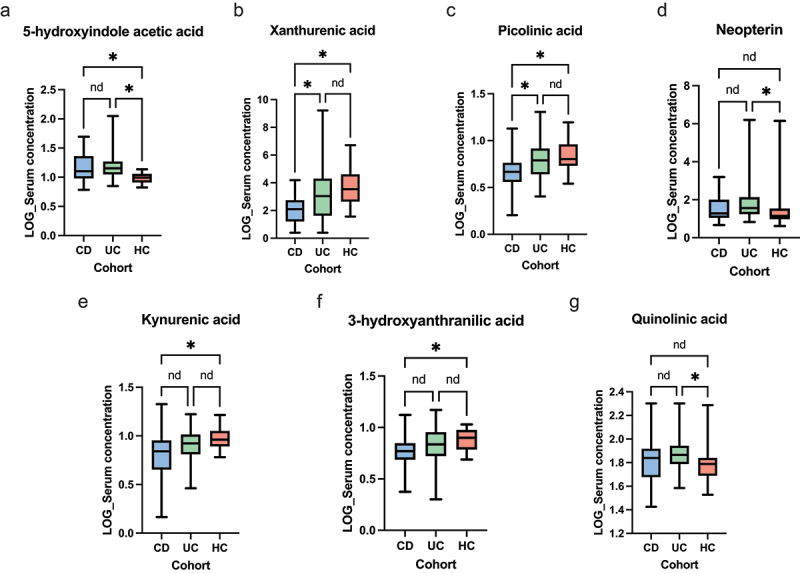
Univariate analyses of serum tryptophan metabolites, inception cohort (a) 5-HIAA (ANOVA *p* < 0.001, CD vs HCs *q* = 0.0057, UC vs HCs *q* < 0.001, CD vs UC *q* = 0.35) (b) Xanthurenic acid (ANOVA *p* = 0.0018, CD vs HCs *q* = 0.0018, UC vs HCs *q* = 0.20, CD vs UC *q* = 0.013). (c) Picolinic acid (ANOVA *p* = 0.0055, CD vs HCs *q* = 0.0092, UC vs HCs *q* = 0.56, CD vs UC *q* = 0.0092). (d) Neopterin (ANOVA *p* = 0.018, CD vs HCs *q* = 0.19, UC vs HCs *q* = 0.015, CD vs UC *q* = 0.19). (e) Kynurenic acid (ANOVA *p* = 0.018, CD vs HCs *q* = 0.015, UC vs HCs *q* = 0.17, CD vs UC *q* = 0.10). (f) 3-HAA (ANOVA *p* = 0.034, CD vs HCs *q* = 0.031, UC vs HCs *q* = 0.24, CD vs UC *q* = 0.12). (g) Quinolinic acid (ANOVA *p* = 0.039, CD vs HCs *q* = 0.23, UC vs HCs *q* = 0.034, CD vs UC *q* = 0.23). The level of significance is represented by *q<0.05, *nd*=no difference.

Targeted LC-MS analyses demonstrated multiple differences between IBD cohorts and HCs in addition to between CD and UC. Serum tryptophan metabolites were markedly different between IBD and healthy controls, additionally notable differences were demonstrated between CD and UC. Serum xanthurenic acid (*q* = 0.0092), picolinic acid (*q* = 0.018), kynurenic acid (*q* = 0.036) and 3-hydroxyanthranilic acid (3-HAA) (*q* = 0.055) were lowest in CD patients compared to both HCs and UC patients. UC patients exhibited higher serum concentrations of neopterin (*q* = 0.036) and quinolinic acid (*q* = 0.055) compared to HCs. Additionally, serum 5-hydroxyindole acetic acid (5-HIAA) was consistently shown to be lowest in HCs compared to CD and UC patients (*q* < 0.001). These data are shown in Figure 4. Multiple differences were noted in serum SCCAs with isobutyrate (*q* = 0.039) and 2-methylbutyrate (*q* = 0.039) increased in UC patients compared to HCs. Serum butyrate (*q* < 0.001) concentration and isovalerate (*q* = 0.053) concentration were lowest in HCs compared to both CD and UC patients. Additionally, UC patients demonstrated a higher serum lactate compared to HCs (*q* = 0.022).

**Figure 5.**
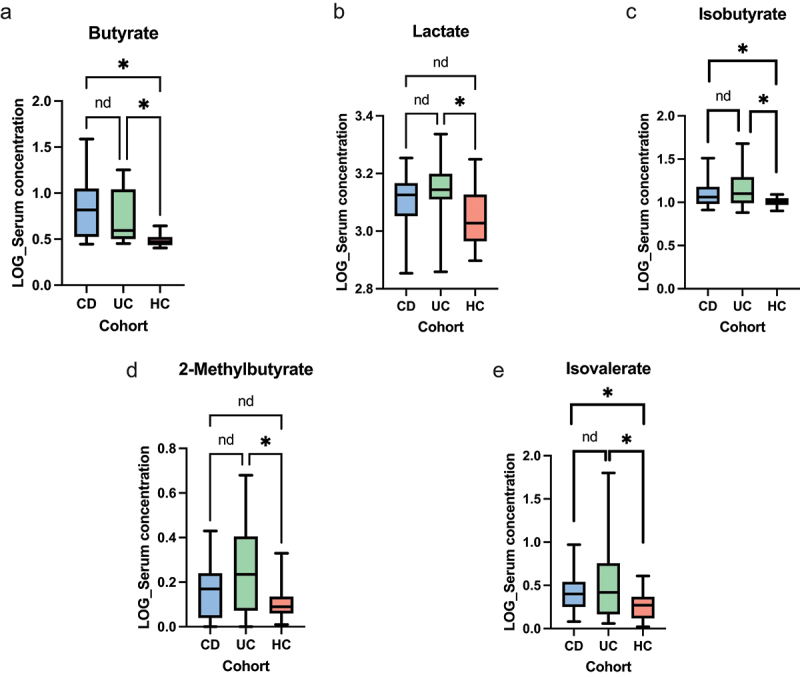
Univariate analyses of serum scfas, inception cohort (a) Butyrate (ANOVA p < 0.001, CD vs HCs *q* < 0.001, UC vs HCs *q* < 0.001, CD vs UC *q* = 0.58). (b) Lactate (ANOVA *p* = 0.0049, CD vs HCs *q* = 0.11, UC vs HCs *q* = 0.0034, CD vs UC *q* = 0.11). (c) Isobutyrate (ANOVA *p* = 0.029, CD vs HCs *q* = 0.054, UC vs HCs *q* = 0.032, CD vs UC *q* = 0.80). (d) 2-methylbutyrate (ANOVA *p* = 0.012, CD vs HCs *q* = 0.24, UC vs HCs *q* = 0.011, CD vs UC *q* = 0.13). (e) Isovalerate (ANOVA *p* = 0.029, CD vs HCs *q* = 0.054, UC vs HCs *q* = 0.032, CD vs UC *q* = 0.80). The level of significance is represented by *q<0.1, *nd*= no difference.

The serum findings are shown in Figure 5.

**Figure 6.**
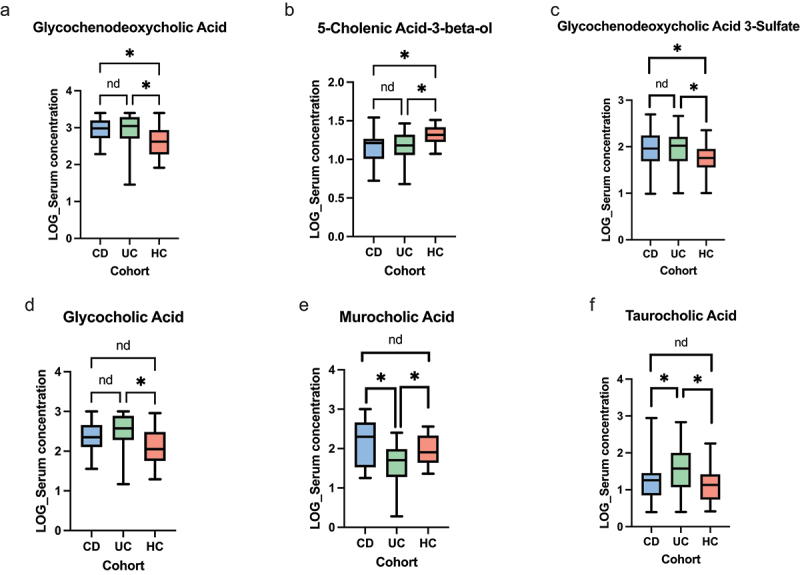
Univariate analyses of serum BAs inception cohort. (a) Glycochendeoxycholic acid (ANOVA *p* = 0.0065, CD vs HCs *q* = 0.014, UC vs HCs *q* = 0.0081, CD vs UC *q* = 0.89). (b) 5-cholenic acid-3-beta-ol (ANOVA *p* = 0.0071, CD vs HCs *q* = 0.011, UC vs HCs *q* = 0.010, CD vs UC *q* = 0.98). (c) Glycochenodeoxycholic acid 3-sulfate (ANOVA *p* = 0.059, CD vs HCs *q* = 0.078, UC vs HCs *q* = 0.072, CD vs UC *q* = 0.90). (d) Glycocholic acid (ANOVA p = 0.0072, CD vs HCs *q* = 0.19, UC vs HCs *q* = 0.0062, CD vs UC *q* = 0.13). (e) Murocholic acid (ANOVA *p* = 0.0082, CD vs HCs *q* = 0.45, UC vs HCs *q* = 0.070, CD vs UC *q* = 0.0099). (f) Taurocholic acid (ANOVA *p* = 0.017, CD vs HCs *q* = 0.58, UC vs HCs *q* = 0.029, CD vs UC *q* = 0.059). The level of significance is represented by *q<0.1, *nd*=no difference.

Serum BA analyses revealed multiple differences between each disease group. Serum glycochenodeoxycholic acid (*q* = 0.014) and glycoursodeoxycholic acid 3-sulfate (*q* = 0.077) concentration were shown to be lowest in HCs compared to CD. A similar trend was observed with UC patients exhibiting a higher concentration of serum glycochenodeoxycholic acid (*q* = 0.0081) and glycoursodeoxycholic acid 3-sulfate (*q* = 0.072) compared to HCs. Serum glycocholic acid was lower in HCs compared to UC patients (*q* = 0.0062) although similar differences were not demonstrated between CD and HCs. Murocholic acid was shown to be the lowest in UC patients compared to both CD (*q* = 0.0099) and HCs (*q* = 0.070). The opposite trend was demonstrated with serum taurocholic acid concentration, as this was highest in UC patients compared to both CD (*q* = 0.059) and HCs (*q* = 0.029). 5-Cholenic acid-3-beta-ol was noted to be highest in HCs compared to both CD (*q* = 0.011) and UC patients (*q* = 0.010). These serum BA findings are shown in Figure 6.

Global profiles using RP modes for fecal and urinary samples noted multiple metabolite concentration differences between HCs and IBD patients in addition to between CD and UC patients. These are listed in Appendix 5 within the Supplementary Material.

### Microbial–metabolite correlation network analyses reveal key differences between IBD and healthy controls

**Figure 7.**
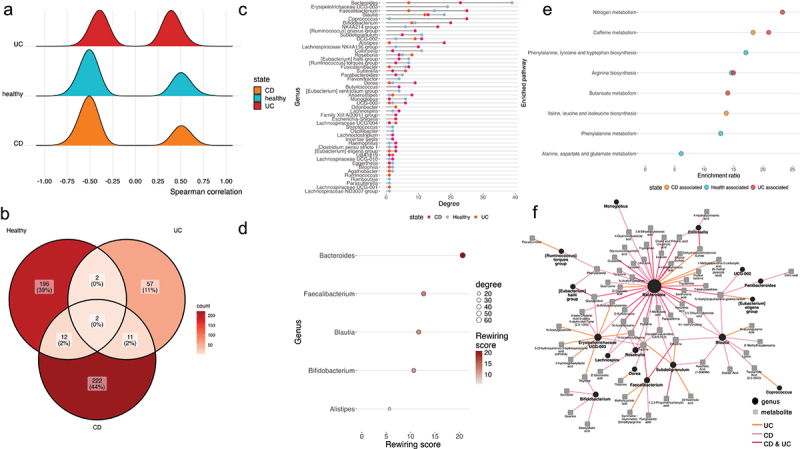
(a) Distribution of correlation values across disease and health states (b) Venn-diagram of strongly positive (r ≥ 0.5) associations across cohorts. (c) Number of strongly positive associations per genera and state (d) Genera with the most variable strongly positive associations across states (e) KEGG pathway enrichment of strongly positive associations across states (f) Associations strongly positively correlated with health and negatively correlated with ulcerative colitis and/or Crohn’s disease.

To further explore and explain the microbiota and metabolomic differences, these data were integrated with correlation network analyses. We observed strong (*r* ≥ 0.5) microbe-metabolite correlations (Figure 7(a)), unique to each condition, with the HCs and CD patients demonstrating the most distinct correlations. The overlaps among groups were small, suggesting a loss of correlations associated with health, demonstrated in Figure 7b. Multiple bacterial genera have strong associations in each cohort, with the genus *Bacteroides, Faecalibacterium*, and *Blautia* correlating with the most metabolites from genera present in all cohorts (Figure 7(c)). *Bacteroides, Faecalibacterium*, and *Blautia* are the genera correlating with the most different metabolites (Figure 7(d)), indicating that the genera with the largest number of correlations are also associated with the most different metabolites across CD and UC patients, and HCs. These strong, positive condition-specific microbe–metabolite correlations correspond to distinct functionally enriched metabolic KEGG pathways (Figure 7(e)). Notably, the metabolites from the microbe–metabolite correlations of UC patients are overrepresented in nitrogen and butanoate metabolism pathways, while CD patients are enriched in valine, leucine, and isoleucine biosynthesis, and both IBD subtypes demonstrate enhanced caffeine metabolism pathways. The healthy controls are characterized by the phenylalanine, tyrosine, and tryptophan biosynthesis and metabolism and alanine, aspartate, and glutamate metabolism pathways, all of which are lacking in the IBD subtypes.

To gain further confidence in the disease relevance of these associations, we analyzed microbe – metabolite correlations, which were strongly positive in health but simultaneously negative in one or more IBD subtypes (Figure 7(f)). KEGG pathway overrepresentation analysis of the metabolites from these matching microbe–metabolite correlations between health and IBD indicated that the taurine and hypotaurine metabolism pathway was lost in both the CD and UC comparisons.

## Discussion

Here we present the analyses of biosamples from a true inception cohort of IBD patients and healthy controls, demonstrating multiple microbial and metabolomic differences between UC, CD, and healthy controls. Additionally, we document microbe–host interactions with network analyses, demonstrating numerous novel microbiome–metabolome correlations that have not previously been well characterized in IBD.

Previous studies have investigated the gut microbiome and metabolome in IBD, with IBD patients characterized by a reduction in microbial diversity,^5^ an enrichment in Proteobacteria,^6^ and a perceived “dysbiotic environment,” as well as a low bile acid concentration compared to a healthy population.^8^ However, previous data have not usually been derived from true inception cohorts, with patients having undergone prior surgery or having been exposed to medical treatment at the time of sample collection. Additionally, prior study sampling was often obtained after endoscopic diagnosis (with the known effects of bowel purgatives upon the gut microbiota and metabolome).^13,14^

Our findings demonstrate notable inter-disease differences between CD and UC patients, including the abundance of Bacteroidetes and numerous differences in serum TRP metabolite concentrations. Our findings corroborate those of Michaudel *et al*. in that TRP metabolite concentrations were increased in healthy controls compared to IBD patients; they have shown that active IBD results in a reduced concentration of kynurenic and xanthurenic acid compared to quiescent disease.^17^ Utilizing correlation network analysis, we observed strong disease-specific microbe-metabolite correlations (*r* ≥ 0.5), with unique interactions primarily in healthy and CD patients and with minimal overlap between cohorts, suggesting a loss of health-associated interactions. Bacterial genera such as *Bacteroides, Faecalibacterium*, and *Blautia* were linked to the most varied metabolites across conditions; these strong positive correlations mapped to distinct metabolic KEGG pathways, with UC patients showing increased nitrogen and butanoate metabolism, CD increased valine, leucine, and isoleucine biosynthesis, and both IBD subtypes enhanced caffeine metabolism. Healthy controls showed enrichment in phenylalanine, tyrosine, tryptophan, and alanine-related pathways. The increase in butanoate metabolism pathways seen in UC patients may inform as to the nature of gut inflammation: previous data have identified butyrate as an inhibitor of neutrophils (isolated from IBD patients) secreting pro-inflammatory cytokines.^53^ The dysregulation of glutamate and tryptophan metabolism pathways, as demonstrated in our cohort, corroborates the findings of Ning *et al*. in their analysis of the fecal microbiome and metabolome in IBD.^12^ Further analysis of shared, but anticorrelating microbe-metabolite pairs revealed health-specific associations negatively correlated with IBD, identifying the taurine and hypotaurine metabolism pathway as activated in both CD and UC (Supplementary Figure S4). This finding correlates with recent data illustrating low serum taurine levels in both CD and UC compared to controls, implicating increased taurine metabolism.^54^ Our novel finding of an increase in caffeine metabolism in IBD patients is of interest: previous murine models of colitis have illustrated the protective role of caffeine,^55^ although to fully understand this interaction, further prospective data with detailed caffeine intake linked to intestinal inflammation are required.

The strengths of our data are numerous. Importantly, samples were obtained prior to endoscopic confirmation of disease. Additionally, as samples were obtained prior to any medical or surgical treatment (which have been shown to alter both microbes and metabolites),^56^ we have been able to present an unadulterated sample cohort. We were able to ensure almost complete sample donation from our entire cohort: 78 out of 80 participants were able to donate a full complement of fecal, serum, and urine samples – this is far higher than in comparable clinical studies. However, we recognize that our work is not without limitations. Our cohort size is smaller than some of the other “multi-omic” studies of IBD^12^, though as discussed above, many of these cohorts have their own limitations, such as samples being taken after purgatives, incomplete sample collection, and from patients who were not treatment-naïve.^7^ The most distinctive feature of our study is the rigorously phenotyped inception cohort that we recruited, with careful sample collection. The relative novelty of inception cohort studies within this field means that no informed power calculations regarding group size could be easily undertaken, although our results will inform such calculations for future, larger studies. Furthermore, while our study recruited from only a single center (arguably with the population living within a close geographical area and experiencing similar environmental exposures, etc.), this was a large center in London, UK, in which there is inherent heterogeneity including population age, ethnicity, and sex, allowing our findings to give insights into diverse IBD populations. We recognize that there is a need to validate our findings in multi-center, diverse populations to confirm the biological relevance of our conclusions. As all our samples were obtained prior to treatment in active IBD, the observed microbial and metabolomic characteristics may at least partly be a proxy of gut inflammation *per se* rather than a predictive signal of IBD: future serial samples during times of clinical or endoscopic flare and comparison with periods of remission may be contributory in this regard. It is of note that recent studies recruiting ‘at risk’ patients prior to the onset of IBD for microbiome analysis found that “microbiota risk scores” for IBD may be independent of the level of intestinal inflammation.^57^ A further study of interest would involve comparison of samples collected from patients with similar demographics to our IBD inception cohort presenting with gastrointestinal symptoms but ultimately being found not to have IBD, but another diagnosis – this may also contribute to deconvolution of the multi-omic signal of what is gut inflammation-derived and what is specifically IBD-related.

Our work combines microbiota and metabolite data in correlation network analyses, thereby linking the findings to truly understand the relationship between the microbe and the host. Further investigation of murine models and human organoids may also serve to enhance our understanding of the underlying biological mechanisms.

In conclusion, this is the first analysis to comprehensively integrate microbiota composition and metabolic function in the study of a truly treatment-naïve inception cohort of patients newly diagnosed with IBD. Using state-of-the-art network biology techniques, we have elucidated novel host–microbe interactions, potentially illustrating pathways related to host–metabolite interactions relevant to IBD pathogenesis that have not been previously explored. Our findings will enable mechanistic studies to probe the impact of these microbial metabolites on aberrant host immune function in IBD and will better inform translational research using interventions including diet and microbial therapeutics such as fecal microbiota transplant to modulate IBD disease course.

This manuscript has been submitted as a preprint.^58^

## Supplementary Material

Supplemental Material

## Data Availability

The raw sequencing data have been deposited at the European Bioinformatics Institute’s (EBI) European Nucleotide Archive ENA database (accessible via https://www.ebi.ac.uk/ena/browser/view/PRJEB86986) under the accession number PRJEB86986.
