# Supplementary material for: Deciphering the microbiome–metabolome landscape of an inflammatory bowel disease inception cohort
Source: Gut Microbes. 2025 Jul 18;17(1):2527863. doi: 10.1080/19490976.2025.2527863 (PMC12279270; doi:10.1080/19490976.2025.2527863)
Supplement: Supplemental Material [file KGMI_A_2527863_SM2976.zip › Gut_Microbes_Revision_Supplementary_Appendices_CLEAN.docx]

**Supplementary Appendices:**

**Appendices**:

1. Additional details regarding metataxonomic analysis:

1a. V1-2 primer sequences.

| Primer name | Primer sequence | Primer ratio |
| --- | --- | --- |
| 28F-YM (Forward primer) | GAGTTTGATYMTGGCTCAG | 4 |
| 28F-Borrellia (Forward primer) | GAGTTTGATCCTGGCTTAG | 1 |
| 28FChloroflex (Forward primer) | GAATTTGATCTTGGTTCAG | 1 |
| 28F-Bifdo (Forward primer) | GGGTTCGATTCTGGCTCAG | 1 |
| 388R (Reverse primer) | TGCTGCCTCCCGTAGGAGT |  |

*1b. Reads generated by metataxonomics:*

During processing of sequencing data, we removed samples with < 7757 reads to remove poor quality reads / samples; however, we included two samples with <10K reads due to the relatively low sample numbers of the unique cohort. We were satisfied that the remaining data is of very good quality, as reflected by the first quantile being 25K read depth.

1. *^1^H NMR setup.*

Urine and fecal water extracts were analyzed using a Bruker 600 MHz AVANCE III NMR spectrometer. The 1D 1H NMR spectra were acquired using a standard one-dimensional pulse sequence, with saturation of the water resonance (noesygppr1d pulse program) during both the relaxation delay (RD = 4s) and mixing time (tm = 10 ms). A standard one-dimensional NOESY pulse sequence was acquired using a pulse width of approximately 13 µs. During this time, a water pre-saturation pulse was applied to suppress the water signal. The two magnetic field z-gradients implemented were applied for 1 ms, the receiver gain was set to 90.5 and acquisition time (ACQ) to 2.73s for all experiments. Each spectrum was acquired using 4 dummy scans, 32 scans, 64 K data points and a spectral window of 20 ppm. Prior to Fourier Transformation, each free induction decay was multiplied by an exponential function corresponding to a line broadening of 0.3 Hz.

For serum, following the recording of the 1D NOESY-presat, one‐dimensional CPMG with water saturation was acquired using the Carr–Purcell–Meiboom–Gill pulse sequence (cpmgpr1d). The acquisition parameters are set up in the same way as the 1D NOESY-presat, with the addition of the spin-echo delay (Delta1/Delta2) that is set at 0.3 ms and the implementation of 128 loops for T2 filter (L4).

2D 1H − 1H J-resolved experiment was also acquired for each sample to detect the J-couplings in the second dimension using the pulse program with suppression of the water resonance during the relaxation delay (jresgpprqf). The acquisition parameters used for this experiment were as follows: 16 dummy scans and 2 scans, 8K points with spectral window of 16.7 ppm for f2 and 40 increments with a spectral window of 78 Hz for f1, incremented delay of 3μs, RD of 2 s and ACQ of 0.41 s. The receiver gain was set to 90.5. A sine-bell apodization function was applied on both dimensions, followed by Fourier transformation, tilting by 45°, and summarization along f1.

The NMR spectra of urine and FW samples were acquired at 300 K, and those for serum were acquired at 310 K.

1. *α diversity comparisons of HCs compared to IBD cohorts.*

| α diversity vs HC | Estimated STD | Error | FDR P value |
| --- | --- | --- | --- |
| Shannon vs UC | -4.4417 | 43.6334 | 0.860681 |
| Shannon vs CD | 20.5496 | 25.2009 | 0.463707 |
| InvSimp vs UC | -3.3224 | 4.8611 | 0.49689 |
| InvSimp vs CD | -3.6301 | 5.3755 | 0.50203 |
| Chao1 vs UC | -4.4417 | 25.2009 | 0.860681 |
| Chao1 vs CD | 20.5496 | 27.8676 | 0.463707 |
| Faith’s PD vs UC | -2.6429 | 3.6969 | 0.4774 |
| Faith’s PD vs CD | 0.1447 | 4.0881 | 0.9719 |

*Abbreviations of Table: InvSimp =Inverse Simpson. STD = Standard deviation. FDR = false discovery rate*

1. *OPLS-DA statistics for ^1^H NMR data*

| OPLS-DA comparison | | R^2^X (cum) | | R^2^Y (cum) | Q^2^ (cum) | | CV-ANOVA P value. | |
| --- | --- | --- | --- | --- | --- | --- | --- | --- |
| Fecal HC vs CD | | 0.168 | | 0.453 | 0.263 | | **0.00355** | |
| Fecal HC vs UC | | 0.391 | | 0.466 | 0.257 | | **0.00341** | |
| Fecal CD vs UC | | 0.242 | | 0.181 | -0.0016 | | 1 | |
| Serum HC vs CD | 0.157 | | 0.869 | | | 0.415 | | **0.0008** |
| Serum HC vs UC | 0.112 | | 0.42 | | | 0.142 | | **0.0199** |
| Serum CD vs UC | 0.295 | | 0.8 | | | 0.224 | | **0.0279** |
| Urine HC vs CD | | 0.185 | | 0.836 | 0.437 | | **0.0006** | |
| Urine HC vs UC | | 0.148 | | 0.743 | 0.153 | | 0.0806 | |
| Urine CD vs UC | | 0.159 | | 0.907 | 0.0806 | | 0.5937 | |

1. *Global LC-MS analyses using RP +ve and -ve modes for feces and RP -ve mode for urine:*

(*Highlighted in yellow if higher in HC compared to IBD subtypes. Highlighted in magenta if higher in CD compared to UC and HCs. Highlighted in blue if highest in CD more than HCs and higher in HCs more than UC. Highlighted in green if lower in HCs compared to IBD subtypes. Highlighted in cyan if higher in HC compared to UC. Highlighted in red if lower in UC compared to CD and HC. Highlighted in grey if higher in CD compared to HC)*

| Metabolite | Biofluid | q value (ANOVA) | Comparison |  |
| --- | --- | --- | --- | --- |
| ***Azelaic Acid*** | Feces | 0.008943 | HC > CD; HC > UC |  |
| ***1,3,7-Trimethyluric acid*** | Feces | 0.008943 | HC > CD; HC > UC |  |
| ***Serotonin*** | Feces | 0.0056889 | HC > CD; HC > UC |  |
| ***1-Methyladenosine*** | Feces | 0.0056889 | HC > CD; HC > UC |  |
| ***N-alpha-acetyl-L-lysine*** | Feces | 0.010159 | HC > CD; HC > UC |  |
| ***Suberic acid*** | Feces | 0.0111 | HC > CD; HC > UC |  |
| ***3-Methylglutaric acid*** | Feces | 0.011196 | HC > CD; HC > UC |  |
| ***Adipic acid*** | Feces | 0.011196 | HC > CD; HC > UC |  |
| ***3,5-Dihydroxybenzoic acid*** | Feces | 0.015839 | HC > CD; HC > UC |  |
| ***N-acetylglutamic acid*** | Feces | 0.017343 | HC > CD; HC >UC |  |
| ***2-Methylglutaric acid*** | Feces | 0.021755 | HC > CD; HC > UC |  |
| ***3HP3HP*** | Urine | 0.055758 | HC > CD; HC > UC |  |
| ***Glutamic acid*** | Feces | 0.06438 | HC > CD; HC > UC |  |
| ***N-Isovaleroylglycine*** | Feces | 0.073789 | HC > CD; HC > UC |  |
| ***4-Hydroxybenzoic acid*** | Feces | 0.073789 | HC > CD; HC > UC |  |
| ***D-Glucuronic acid*** | Feces | 0.015834 | CD > HC; CD > UC |  |
| ***Glycylproline*** | Feces | 0.017343 | CD > HC; CD > UC |  |
| ***Malic acid*** | Feces | 0.021755 | CD > HC; CD > UC |  |
| ***Adenosine 3’,5’-cyclic monophosphate*** | Urine | 0.063054 | CD > HC; CD > UC |  |
| ***5-hydroxyindole sulphate*** | Urine | 0.081918 | CD > HC; CD > UC |  |
| ***Xanthurenic acid*** | Feces | 0.088469 | CD > HC; CD > UC |  |
| ***Glycoursodeoxycholic acid*** | Feces | 0.073789 | CD > HC; HC > UC |  |
| ***Guanine*** | Feces | 0.088469 | CD > UC; HC > UC |  |
| ***Riboflavin (vit B2)*** | Feces | 0.088469 | CD > UC; HC > UC |  |
| ***Na-acetyl-L-arginine*** | Urine | 0.055758 | CD > UC; HC > UC |  |
| ***Isobutyrylglycine*** | Urine | 0.063054 | CD > UC; HC > UC |  |
| ***2,5-Dihydroxybenzoic acid*** | Urine | 0.063054 | CD > UC; HC > UC |  |
| ***Hypoxanthine*** | Urine | 0.089285 | CD > UC; HC > UC |  |
| ***Xanthine*** | Urine | 0.089285 | CD > UC; HC > UC |  |
| ***Dehydroepiandrosterone Sulphate*** | Feces | 0.024999 | CD > HC; UC > HC |  |
| ***Suberic acid*** | Urine | 0.04848 | CD > HC; UC > HC |  |
| ***23-Norcholic acid*** | Urine | 0.04848 | CD > HC; UC > HC |  |
| ***Azelaic Acid*** | Urine | 0.04848 | CD > HC; UC > HC |  |
| ***Dopamine 3-O-sulphate*** | Urine | 0.063054 | CD > HC; UC > HC |  |
| ***Glutamine*** | | Feces | 0.085147 | CD > HC; UC > HC |
| ***Hypoxanthine*** | | Feces | 0.085147 | CD > HC; UC > HC |
| ***Tryptophan*** | | Feces | 0.088469 | CD > HC; UC > HC |
| ***1,7-Dimethyluric acid*** | Feces | 0.096193 | HC > UC |  |
| ***3-Hydroxyhippuric acid*** | Urine | 0.063054 | HC > UC |  |
| ***Succinylaminoimidazole carboxamide riboside*** | Urine | 0.063054 | CD > HC |  |
